# Antenatal care follow-up was significantly associated with a higher probability of high dietary diversity score among pregnant women in okra-producing areas of western Ethiopia: proportional odds model

**DOI:** 10.29219/fnr.v67.9608

**Published:** 2023-07-28

**Authors:** Efrem Negash Kushi, Tefera Belachew, Dessalegn Tamiru

**Affiliations:** 1 Department of Public Health, College of Health and Medical Science, Mettu University, Mettu, Ethiopia; 2 Departments of Nutrition and Dietetics, Public Health Institute, Jimma University, Jimma, Ethiopia

**Keywords:** dietary modification, okra consumption, maternal nutrition, ordinal logistic regression, Ethiopia

## Abstract

**Background:**

Dietary diversity is important for pregnant women since it has been associated with nutrient adequacy. It is very crucial to ensure optimal fetal health and development. There is no evidence at the community level on the magnitude of dietary diversity and its predictors among pregnant women in okra-producing areas of western Ethiopia.

**Objective:**

This study aimed to assess the level of dietary diversity and its associated factors among pregnant women.

**Design:**

A community-based cross-sectional study was employed among randomly selected 224 pregnant women in western Ethiopia. An interviewer-administered questionnaire was used to collect the data. The qualitative open 24-h recall was used to assess the level of dietary diversity. Ordinal logistic regression analyses were used by SPSS version 25. An adjusted proportional odds ratio along with a 95% confidence interval [CI] was computed to measure the strengths of the association at a *P* ≤ 0.05.

**Result:**

This study revealed that more than one-fourth, 64 (28.6%), of the respondents were found to have high dietary diversity scores. Antenatal Care [ANC] visits (Adjusted Odds Ratio [AOR] = 2.10, [95% CI: 1.13, 3.90], *P* = 0.01), changing food intake (AOR = 2.97, [95% CI: 1.16, 3.67], *P* = 0.002), and being food secure household (AOR = 2.63, [95% CI: 1.38, 5.00], *P* = 0.003) were significantly associated with a higher probability of having high dietary diversity score. However, lack of formal education (AOR = 0.34, [95% CI: 0.61, 0.89]) was inversely associated with the probability of having high dietary diversity.

**Conclusion:**

More than half of pregnant women in western Ethiopia were found to have low dietary diversity. Therefore, ANC follow-up, dietary modification, and promotion of frequent use of wild edible plants (okra) to maintain household food security were very crucial.

## Popular scientific summary

- A diversified diet that includes locally available edible plants has an important role for pregnant women.
- Okra is a cost-effective and economically affordable natural source that reduces malnutrition in resource-limited settings.
- This study emphasized the need for agricultural and nutritional policy attention to wild edible plants like okra.
- Identifying community-based evidence related to the dietary diversity of pregnant women is crucial to design sustainable evidence-based nutritional interventions to maintain maternal nutrition.

Dietary diversity has been defined as the number of different food groups that are consumed over a specific reference period (1). It is important for pregnant women since it has been associated with nutrient adequacy (2, 3). Globally, micronutrient deficiencies are common among pregnant women (4). For this reason, a diversified diet is critical to ensure an adequate intake of micronutrients and to maintain optimal maternal nutrition (5, 6). Thus, dietary diversity during pregnancy is very crucial to ensure optimal fetal health and development (7–9).

In developing countries, the maternal diet is monotonous (plant-based or cereal-based) with little consumption of animal source foods as a result of poverty (10). Accordingly, 45% of pregnant women in the western region of Nepal did not consume a diverse diet (11). Similarly, 89% of the pregnant women in Pakistan had medium dietary diversity, while only 5% showed low and high dietary diversity (12). In line with this, 79.9% of pregnant women in Ghana met the minimum dietary diversity (13), while only 32.4% of pregnant women in rural Malawi met the minimum dietary diversity (14).

The pooled prevalence of inadequate dietary diversity was 53%, which is high at the national level in Ethiopia (15). On the other hand, 51.7% of rural South West Ethiopia (16), 31.4% of North East Ethiopia (17), 29.46% of eastern Ethiopia (18), and 42.6% of pregnant women in southern Ethiopia (19) had adequate dietary diversity. However, 51% in South West Ethiopia (20), 25.4% in Central Ethiopia (21), 51.6% in Addis Ababa, Ethiopia (22), and 84.4% of pregnant women in south-central Ethiopia (23) had minimum dietary diversity. Furthermore, 61.2 and 38.8% of pregnant women in the Tigray region, Ethiopia, had high and low dietary diversity scores, respectively (24).

In developing countries including Ethiopia, the dietary diversity of pregnant women is associated with household wealth index, household food insecurity, family size (18, 19, 25, 26), age of pregnant women (27), anemia (7), education status of women, occupational status of women, dietary diversity knowledge (8, 16, 28, 29), and favorable attitude (11).

One of the most common staple diets for the indigenous Community of Asossa District, western Ethiopia, is okra. In line with this, there is also evidence which indicated that okra was first found in Ethiopia and later distributed to other parts of the world while gaining popularity in the West (28).

Okra contributes an important role in the human diet, especially important for pregnant women (30, 31). It has different roles for pregnant women due to its high bioavailable iron, which ranges from 8.33 to 20.29 mg/100 g (30, 32). Okra seed flour, leaves, and pods of okra contain considerable amounts of iron which are very crucial in the prevention of iron deficiency anemia during pregnancy (25, 27, 29, 33). Furthermore, okra also contains high (88 mcg/100 g) folate which is used to prevent neural tube defects and promote the normal development of the placenta (26, 34). For this reason, okra is used to maintain the micronutrient adequacy of pregnant women and improves the dietary diversity of pregnant women. Generally, okra is a good source of both essential macro- and micronutrients (35, 36). Therefore, okra is a cost-effective and economically affordable food source which is very crucial for improving dietary diversity and maternal nutrition in resource-limited settings (31, 37, 38).

A monotonous diet of pregnant women has a crucial impact on their nutritional status, which in turn affects their health and pregnancy outcomes (39). However, adequate nutritional intake of both macronutrients and micronutrients is essential for pregnant women (40). Food insecurity, which leads to the consumption of monotonous diets as well as poor-quality diets, remains a challenge for pregnant women (41). For this reason, different global nutrition interventions have focused on improving the dietary diversity and nutritional status of pregnant women and children in the first 1,000 days and then improving their health (42). Likewise, the government of Ethiopia has made tremendous efforts to reduce maternal malnutrition through multisectoral collaboration and dietary modification by promoting locally available foods to maintain the dietary diversity of pregnant women. However, pregnant women in rural areas depend on a monotonous diet, and the burden of undernutrition among pregnant women is still high (43 – 45).

Even though there was evidence about the dietary diversity of pregnant women in Ethiopia, most of them were institutional-based. In line with this, there is no evidence of the magnitude of dietary diversity and its associated factors among pregnant women in western Ethiopia. Therefore, identifying community-based evidence related to the dietary diversity of rural pregnant women is very crucial to design sustainable evidence-based nutritional intervention strategies to maintain maternal nutrition. Therefore, this study aimed to assess the level of dietary diversity and its associated factors among pregnant women in okra-producing areas of western Ethiopia.

## Materials and methods

### Study design and setting

A community-based cross-sectional study design was employed from June 1 to July 30, 2020. This study was conducted in the Asossa zone, which is located in the Benishangul Gumuz regional state of western Ethiopia. The indigenous communities in the region are Berta, Gumuz, Shinasha, Maho, and Komo. The climatic condition of the Asossa zone is tropical (Benishangul Gumuz Regional Health Beruae District Plan of 2019, unpublished work).

### Source and study population

All households with pregnant women of Berta community who live in the Asossa zone were the source population. In addition to this, all households of pregnant women of Berta community and those in their first trimester who have been included in the sample of selected kebeles were the study population. In line with this, households with at least one pregnant woman were included. For more than one eligible woman in the selected households, the one who was responsible for family care was considered for this study.

### Inclusion and exclusion criteria

Pregnant women of the Berta community who are permanent residents (who live in the study area for a duration of more than or equal to 6 months), who are in the first trimester of pregnancy (having symphysis-pubis fundal height [SFH] measurement of 6 cm or the fundus at the level of the pubic bone), and who use the okra plant as their food source were included in this study. Those Berta community women who are critically ill, with acute health problems based on their chief complaints (self-report) such as those having abdominal cramps, fever, diarrhea, and headache, were excluded. Furthermore, those pregnant women who complained of chronic health problems were excluded as well. Those who were not willing to give consent and second- and third-trimester pregnancies were excluded. Finally, those pregnant women having the aforementioned health problems were recommended to visit the nearest health facilities for diagnosis and treatment.

### Sample size determination

The sample size was calculated by using G*Power 3.0 based on the following assumptions: Z tests of difference between two independent proportions (p1 = 0.5 and p2 = 0.6), power of 80%, a margin of error 5% with 95% confidence level. Thus, the sample size by considering the design effect of 2 was 204 pregnant women. Finally, by considering the 10% non-response rate, 224 pregnant women of the Berta community were selected.

### Sampling procedure

Kebeles (districts) were selected by a simple random sampling method. The identification and registration of women with known pregnancies were performed from each household using the pregnancy screening checklist. The screening checklist consisted of six items with ‘Yes’ or ‘No’ responses – (1) questions that asked about delivery and breastfeeding in the last 6 months, (2) delivery in the last 4 weeks, (3) the menstrual period in the last 7 days, (4) abortion, or miscarriage in the last 7 days, (5) sexual abstinence since the last menstrual period, and (6) the current use of contraceptives. In resource-limited settings, there is a lack of access to reliable pregnancy conformation such as ultrasound. Therefore, in addition to the pregnancy screening checklist, the urine of the woman who was suspected as pregnant was tested for confirmation using a WHO rapid test kit (HUMAN Gesellschaft für Biochemica und Diagnostica mbH, Wiesbaden, Germany) (46). Finally, eligible first-trimester pregnant women in selected kebeles were selected by a simple random sampling technique.

SFH was used to identify the trimester of pregnancy. SFH was used for the gestational age assessment of pregnant women in resource-limited settings. It is the distance measured from the top of the symphysis pubis to the depression in front of the pad of the middle finger marking the top of the uterine fundus, in the midline of the woman’s abdomen. Measures were rounded to the nearest centimeter (47). Then those pregnant women having SFH measurement of 6 cm or the fundus at the level of the pubic bone were considered to be at the first trimester of pregnancy.

### Data collection tool and procedure

The data were collected using a pretested, interviewer-administered structured questionnaire while the blood sample for each woman was analyzed for hemoglobin concentration in the lab.

### Hemoglobin concentration

This was measured by taking a finger-prick blood sample of each woman using a HemoCue Hb 301 (HemoCue AB, Angelholm, Sweden). Then, a prick was made on the tip of the middle finger after the site was cleaned with disinfectant. Three drops of blood from the left ring finger were collected. The first and second drops of blood were wiped away. Then, the third drop was collected to fill the micro cuvette which was placed in the cuvette holder of the device for measuring hemoglobin concentration and the results were recorded and treated as a covariate in ordinal logistic regression. Finally, the hemoglobin level below the cut of 11.0 g/dL in pregnant women was taken for anemia (48).

### Dietary practices

It was evaluated by eight structured questionnaires which had internal consistency (Cronbach’s alpha of 0.82). Each question was given one mark if the answers were healthy for dietary practices and zero if the responses were unhealthy for dietary practices. Finally, pregnant women were classified to have poor dietary practices coded as ‘1’ if they correctly answered < 75% of practice questions and good dietary practices coded as ‘2’ if they correctly answered ≥ 75% of questions (49).

### Dietary diversity

The Women’s Dietary Diversity Score (WDDS) reflects the probability of micronutrient adequacy of the diet and therefore food groups included in the score put more emphasis on micronutrient intake than on economic access to food (50). For this reason, nine food groups were proposed to calculate the score – (1) starchy staples (a combination of cereals, white roots, and tubers), (2) dark green leafy vegetables, (3) other vitamin A–rich fruits and vegetables (a combination of vitamin A–rich vegetables and tubers, and vitamin A–rich fruits), (4) other fruits and vegetables, (5) organ meat, (6) meat and fish, (7) eggs, (8) legumes, nuts, and seeds, and (9) milk and milk products (50).

The 24-h recall data are widely recognized as a key dimension of diet quality which is quick, can reach a lot of people, and may be used remotely. It was assessed by a qualitative open 24-h recall: the enumerator asked a series of standard probing questions to help the women recall all foods and beverages consumed the previous day and night. He/she also probes for the main ingredients in mixed dishes. Specifically, the recall period covers from when the respondent awoke the previous day through the day and night for 24 h.

Each food or beverage that the respondent mentioned can be circled or ticked on a predefined list. Foods not already included on the predefined list can be either classified by the enumerator into an existing predefined food group or recorded in a separate place on the questionnaire and coded later into one of the predefined nine food groups (51). The dietary diversity score was computed and divided into tertials. Finally, the level of dietary diversity was created and coded as ‘low = 1, medium = 2, and high = 3’ dietary diversity of pregnant women.

### MUAC

It was assessed by using an adult non-stretchable mid-upper arm circumference (MUAC) measuring tape on the left arm while the left arm was relaxed along the body trunk. The reading was taken to the nearest 0.1 cm (52). This was treated as a covariate in ordinal logistic regression.

### Knowledge of diversified diet

A total of 12 items were used to measure the knowledge of participants for the use of a diversified diet. The items had internal consistency (Cronbach’s alpha of 0.87). The scores of knowledge-related questions were converted into tertials. Measurements of those who had the highest tertial were categorized as good knowledge and coded as ‘2’, while the two lower tertials were combined and labeled as poor knowledge which was coded as ‘1’.

### Attitude toward diversified diet

Participants’ attitude was measured using a five-point Likert scale (Cronbach’s alpha of 0.89). The final scores of a five-point Likert scale were computed and then converted into tertials. Those who had the highest tertial were categorized and coded as having a ‘favorable attitude = 1’, while the two lower tertials were combined and labeled as ‘unfavorable attitudes = 0’.

### Household food insecurity

To assess household food insecurity, a series of nine questions were presented to pregnant women. The questions were asked during the past month to assess the experiences of a pregnant woman during a lack of access to food. The ‘Yes’ responses were coded as 1 and the ‘No’ responses as 0. The responses were computed to produce indices of household food insecurity. The final score was divided into tertials and dummy variables were created. Participants grouped in the highest tertial were labeled and coded as ‘food insecure = 1’, while the two lower tertials were combined and labeled as ‘food secure = 0’ (53).

### Frequency of okra consumption

It was assessed by pretested interviewer-administered questionnaires. Dummy variables were created by categorizing participants who consumed okra daily as coded 1 while those who consumed sometimes were coded 0.

### Data quality control

Data quality was ensured by training data collectors and supervisors on the purpose of the research, use of the data collection tools, and the validation of measuring instruments. Furthermore, the English version questionnaires were translated into the native language (Rutanegna) of the respondents by language experts and then back to the English version to check its consistency. Likewise, the collected data were checked out for completeness, accuracy, and clarity. Moreover, a pre-test of instruments for data collection was done among 45 pregnant women in the Bambasi district, western Ethiopia. Finally, data cleanup and cross-checking were also done before data analysis.

### Ethical considerations

The study protocol was approved by the Institutional Review Board of Jimma University, Institute of Health, and all participants provided written informed consent. Therefore, the study participants were informed about the research and their right to participation (right to decline participation at any time they feel to do so). Finally, those with severe health problems were informed to visit public health facilities.

### Data processing and analysis

Collected data were entered into Epi-data version 3.1 and then exported to SPSS version 25 for analysis. Furthermore, normally distributed continuous variables were presented as means and standard deviations. The ordinal logistic regression model (proportional odds model [POM]) was used for data analysis after all important assumptions were checked. The POM (cumulative logit model) is the most widely used ordinal logistic regression model in practice or epidemiological studies (54, 55).

Multicollinearity was checked using a variance inflation factor (VIF) and all independent variables had a VIF of less than 10. Finally, outliers were checked by Cook’s distance statistic and there were no outliers (Cook’s distance < 1).

The ordinal logistic regression was run by a combination of two procedures both having their downside procedure. The first procedure, Polytomous Universal Model [PLUM], was used to compute parameter estimates of each predictor, model goodness-of-fit statistics, save predicted probabilities, and test POM. However, this procedure does not automatically compute the antilogarithms of the regression coefficients. For this reason, the second procedure GENLIN (generalized linear model) was used to compute the antilogarithms of the regression coefficients of each predictor variable.

The proportional odds assumption essentially states that the relationship between the independent variable and dependent variable is constant irrespective of which groups are being compared on the dependent variable (56). Therefore, a significant test of parallel lines would mean that probability of falling to a higher category does not vary across categories on the dependent variable for the predictors.

The proportional odds assumption was tested using the test of parallel lines (*P* = 0.060), indicating that the effects of the predictors were the same across the levels of dietary diversity. The Pearson chi-square goodness-of-fit test indicated that the model adequately describes the data (*P* = 0.999). In line with this, model fitting information indicated that there is a significant improvement in fit as compared to the null model (*P* = 0.000), hence the model was showing a good fit. Furthermore, Nagelkerke R-square (0.368) indicated that there had been a 36.8% improvement in the prediction of dietary diversity based on the predictors in comparison to the null model.

The bivariable and multivariable POM was fitted to identify predictors of higher dietary diversity. All variables with a *P*-value ≤ 0.25 in the bivariable analysis were fitted into the multivariable analysis to control confounding effects. An adjusted proportional odds ratio with a 95% confidence interval [CI] was used to evaluate the strength of the statistical association between the explanatory and outcome variables. Finally, variables with *P*-values < 0.05 in the multivariable analysis were considered to be statistically significant.

## Results

### Sociodemographic characteristics of the respondents

A total of 224 pregnant women participated in the study with a response rate of 100%. The mean (standard deviation [SD]±) age of the respondents was 25.29 (5.63) years. Eighty-four (37.5%), 71 (31.7%), and 69 (30.8%) pregnant women were categorized in the age group of 16–22, 23–27, and 28–40 years, respectively. Almost more than half (57.1%) of the respondents were able to read and write. Likewise, the majority (96.4%) of them were married in terms of their marital status. Almost all (97.8) of the study participants were Muslim followers. Only 1.3% of respondents were government employees, while 14.7% of the respondents were non-governmental organization (NGO) workers. Regarding the head of household, 98.7% of the household was headed by the husband (Table 1).

**Table 1:** Sociodemographic characteristics of study participants (*n* = 224), western Ethiopia, 2020

Variables (category)	Level of dietary diversity			Total	Tests	
	Low	Medium	High	*N* (%)	X^2^	*P*
**Educational status**						
Unable to read and write	69 (35.4)	12 (6.2)	15 (7.6)	96 (49.2)	16.03	< 0.001
Able to read and write	60 (26.8)	19 (8.5)	49 (21.8)	128 (57.1)		
**Marital status**						
Married	122 (54.5)	30 (13.4)	64 (28.6)	216 (96.4)	3.83	0.43
Divorced/widowed	7 (3.1)	1 (0.4)	0 (0.0)	8 (3.5)		
**Religion**						
Protestant	0 (0.0)	1 (0.4)	4 (1.8)	5 (2.2)	7.82	0.02
Muslim	129 (57.6)	30 (13.4)	60 (26.8)	219 (97.8)		
**Occupation**						
Student	4 (1.8)	1 (0.4)	3 (1.3)	8 (3.6)	26.73	0.003
Housewife	75 (33.5)	22 (9.8)	56 (25.0)	153 (68.3)		
Daily laborer	15 (6.7)	0 (0.0)	4 (1.8)	19 (8.5)		
Merchant/governmental employee	10 (4.4)	1 (0.4)	0 (0.0)	12 (4.9)		

### Pregnancy and related characteristics of the study participants

Regarding the number of pregnancies, 37.1% of them had two to three pregnancies. In line with this, 69 (30.8%) and 72 (32.1%) of the respondents had been one and four to eight times pregnant, respectively. Concerning the interpregnancy interval, almost half (56.7%) of them had a 1-year interval while the rest had 2 to 5 years (Table 2).

**Table 2:** Pregnancy and related characteristics of respondents (*n* = 224), western Ethiopia, 2020

Variables (category)	Level of dietary diversity			Total	Tests	
	Low	Medium	High	*N* (%)	X^2^	*P*
**Abortion**						
Yes	13 (5.8)	1 (0.4)	6 (2.7)	20 (8.9)	1.46	0.48
No	116 (51.8)	30 (13.4)	58 (25.9)	204 (91.1)		
**ANC visits**						
Yes	59 (26.3)	12 (5.4)	34 (15.2)	105 (46.9)	1.90	0.38
No	70 (31.3)	19 (8.5)	30 (13.4)	119 (53.1)		
**Took iron/folate**						
Yes	78 (34.8)	22 (9.8)	46 (20.5)	146 (65.2)	2.98	0.22
No	51 (22.8)	9 (4.0)	18 (8.0)	78 (34.8)		

### Dietary diversity practices

All the study participants (100%) have utilized okra and 62.5% of them were consuming it daily. This study showed that 36.6, 38.8, and 83.5% of pregnant women used fruits, vegetables, and energy-source food daily, respectively. In addition to this, 33 and 13.4% of the respondents changed their food intake and took extra food during pregnancy, respectively. More than one-fifth (21%) of pregnant women avoided at least one food during their pregnancy due to cultural factors, while 13.4% of them took one extra food. Similarly, 43.3% of the total respondents have skipped meals during their pregnancy, and of this, 39.7, 5.4, and 0.4% of them skipped breakfast, lunch, and dinner, respectively. Likewise, 129 (57.6%), 31 (13.8%), and 64 (28.6%) of the pregnant women were found to have low, medium, and high dietary diversity, respectively. Moreover, 152 (67.9%) pregnant women had an unfavorable attitude toward a diversified diet (Table 3).

**Table 3:** Dietary practice and related characteristics of respondents (*n* = 224), western Ethiopia, 2020

Variables (category)	Level of dietary diversity			Total	Tests	
	Low	Medium	High	*N* (%)	X^2^	*P*
**Knowledge of diversified diet**						
Poor	92 (41.1)	33 (14.7)	26 (11.6)	151 (67.4)	23.97	< 0.001
Good	5 (2.2)	9 (4.0)	59 (26.3)	73 (32.6)		
**Frequency of okra consumption**						
Sometimes	59 (26.3)	17 (7.6)	8 (3.6)	84 (37.5)	24.77	< 0.001
Daily	70 (31.3)	14 (6.3)	56 (25.0)	140 (62.5)		
**Food insecurity status**						
Secure	74 (33.0)	23 (10.3)	51 (22.8)	148 (66.1)	10.56	0.005
Insecure	55 (24.6)	8 (3.6)	13 (5.8)	76 (33.9)		

The association between the levels of dietary diversity and knowledge of pregnant women toward a diversified diet was checked by the chi-square goodness-of-fit test. Thus, the null hypothesis was rejected (x^2^ (2) = 23.97, *P* < 0.001), which indicated that there was a statistically significant difference between the knowledge of pregnant women and their level of dietary diversity. For this reason, of the total 32.6% of pregnant women who had good knowledge of a diversified diet, 26.3% of them had a high dietary diversity score. However, of the total pregnant women who had poor knowledge of a diversified diet (67.4%), only 11.6% of them had a high dietary diversity score (Table 3).

### Nutritional status and related characteristics of respondents

**Fig. 1:**
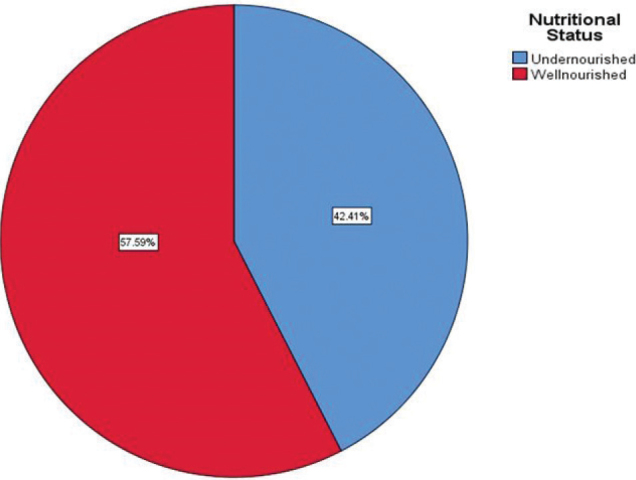
Nutritional status of respondents (*n* = 224), western Ethiopia, 2020.

The finding of this study revealed that 54 (24.1%) of pregnant women had hemoglobin concentration < 11 g/dl (anemic). Furthermore, three-fourth (72.22%) of pregnant women have received iron folate supplementation during pregnancy. In addition to this, 62.9% of the respondents had an unfavorable attitude toward a diversified diet (Fig. 1).

### Factors associated with dietary diversity of pregnant women

Educational status, marital status, ANC visits, changing food intake, food insecurity, and abortion were selected as candidate variables (at *P*-value < 0.25) for the final model. In the final multivariable POM, the variables such as hemoglobin concentration of pregnant women (AOR = 1.51, [95% CI: 1.23, 1.84], *P* < 001), ANC visits (AOR = 2.10, [95% CI: 1.13, 3.90], *P* = 0.01), changing food intake during pregnancy (AOR = 2.97, [95% CI: 1.16, 3.67], *P* = 0.002), and being food secure (AOR = 2.63, [95% CI: 1.38, 5.00], *P* = 0.003) were significantly associated with a higher probability of being classified in high dietary diversity at *P*-value < 0.05. However, those pregnant women with educational status of unable to read and write (AOR = 0.34, [95% CI: 0.61, 0.89]) were significantly associated with a lower probability of being classified in high dietary diversity at *P*-value < 0.05 (Table 4).

**Table 4:** Proportional odds model for predictors of dietary diversity among pregnant women (*n* = 224), western Ethiopia, 2020

Variables (category)	Estimates (B)	COR (95% CI)	AOR (95% CI)	*P*
**Educational status**				
Unable to read and write	−1.07	1.23 (0.55, 1.65)	0.34 (0.61, 0.89)	< 0.001**
Able to read and write		1.00	1.00	
**Marital status**				
Married	4.73	3.32 (16.68, 21.08)	5.7 (0.98, 3.67)	0.98
Divorced	5.98	6.04 (17.25, 18.45)	6. 54 (0.34, 4.76)	0.36
Widowed		1.00	1.00	
**Abortion**				
No	0.002	0.81 (−1.13, 0.70)	1.02 (0.35, 2.79)	0.99
Yes		1.00	1.00	
**Changing food intake**				
Yes	1.09	0.36 (−1.61, −0.42)	2.97 (1.16, 3.67)	0.002*
No		1.00	1.00	
**Food security status**				
Food secure	0.96	2.58 (0.37, 1.54)	2.63 (1.38, 5.00)	0.003*
Food insecure		1.00	1.00	
**ANC visits**				
Yes	0.74	1.19 (−0.32, 0.70)	2.10 (1.13, 3.90)	0.01*
No		1.00	1.00	
Hemoglobin concentration	0.41	1.48 (0.22, 0.56)	1.51 (1.23, 1.84)	< 0.001**

Likelihood ratio chi-square (9) = 64.05, *P*-value < 0.001.

* Significance at *P*-value < 0.05;

** significance at *P*-value ≤ 0.001.

## Discussion

According to this study, nearly more than half, 129 (57.6%), of the pregnant women were found to have low dietary diversity. This proportion was high in comparison to that of the pooled prevalence of inadequate dietary diversity (53%) at the national level in Ethiopia and others (15, 11, 24). In line with this, 151 (67.4%) of the respondents had poor knowledge of a diversified diet. Knowledge of diversified diet and level of dietary diversity was highly correlated (x^2^ = 23.97, *P* < 0.001). Thus those who did not know about diversified diets may not consume different food groups or are based on monotonous diets. For this reason, more than half of the respondents had a low level of dietary diversity. Even though it was not statistically significant, there was evidence in which knowledge of a diversified diet was found to be a predictor of dietary diversity of pregnant women (16, 22).

The finding of this study revealed that 64 (28.6%) pregnant women were found to have high dietary diversity. This was lower than the finding (61.2%) of a study conducted in the Tigray region, Ethiopia, (24) and higher than other study findings (12). This discrepancy might be due to the difference in the study settings, socioeconomic and cultural factors that may hinder maternal nutrition, the difference in the sample size, and the dietary practice of pregnant women across different cultures.

This study also revealed that the educational status of pregnant women was highly significantly associated with their level of dietary diversity. For this reason, the odds of having high dietary diversity were 0.34 times lower for those pregnant women not able to read and write (AOR [95% CI]: 0.34 [0.61, 0.89]) as compared to their counterparts. This indicated that only 25% of them had the probability to be classified as having high dietary diversity. This might be because those who were unable to read and write might not get access to different nutritional-related information from leaflets, bulletins, and other sources. Likewise, they might not know the importance of a diversified diet, nutrition during pregnancy, and its impact on the fetus. This was supported by similar findings as those pregnant women with higher education had greater odds of having high dietary diversity (3, 16).

In this study, we observed that the odds of having high dietary diversity were 2.97 times greater for those pregnant women who changed their dietary intake during pregnancy (AOR [95% CI]: 2.97 [1.16, 3.67]) as compared to those who did not change their dietary intake. This indicated that dietary modification has a crucial impact on the dietary diversity of pregnant women. Thus pregnant women who changed their dietary intake might use different food groups and hence improve their level of dietary diversity.

The odds of having high dietary diversity was 2.63 times greater for those pregnant women who had household food security (AOR [95% CI]: 2.63 [1.38, 5.00]) as compared to those who had household food insecurity. This indicated that those households who had food security might use diversified food and also might have access to different food groups. During food insecurity, pregnant women might have used different coping strategies such as skipping meal patterns, consumption of monotonous diet, and low intake of macro- and micronutrients, which lead them to a lack of diet diversity. This finding was supported by different evidence as food security status was a significant predictor of dietary diversity of pregnant women (13, 14, 23).

The current study indicated that there was also a positive association between the ANC service and having high dietary diversity among pregnant women. For this reason, those pregnant women who visited ANC 2.10 times had high dietary diversity (AOR [95% CI]: 2.10 [1.13, 3.90]) as compared to their counterparts. This finding was consistent with different evidence (45, 57, 58). This might be due to those pregnant women who get ANC having access to nutritional education in which they obtained awareness regarding the importance of a diversified diet and the role and impact of maternal nutrition on child growth and development. In line with this, they might change their behavior related to their dietary intake, dietary modification, and cultural factors affecting their food intake. For this reason, pregnant women who attended ANC might have increased nutritional knowledge, which might have improved their dietary diversity. Thus, increased nutrition knowledge was associated with improved dietary diversity (59).

The finding of the current study also indicated that the odds of having high dietary diversity were 1.51 times greater for each 1 g/dl increase in hemoglobin concentration (AOR [95% CI]: 1.51 [1.23, 1.84]) of pregnant women. This might be because those pregnant women who had high dietary diversity have micronutrient adequacy like iron which increases hemoglobin concentration. This indicated that the consumption of a diverse diet is very crucial to ensure an adequate intake of micronutrients. There were findings that pregnant women with low hemoglobin concentration were vulnerable to having a low dietary diversity (60 – 63).

### Strengths and limitations of the study

It is a community-based cross-sectional study that makes it a representation of the target population. In line with this, it considers the indigenous community within their agroecology, which further helps to generalize the findings to the study area. However, since the dietary diversity score was based on self-report, it might be subjected to recall bias and also seasonal variations for the dietary diversity were not considered due to the cross-sectional nature of the study. Furthermore, it does not show the temporal or causal effect relationships between different factors and the outcome variable.

## Conclusion

More than half of the pregnant women in western Ethiopia were found to have low dietary diversity. ANC visits, changing food intake during pregnancy, and household food security status were significantly associated with a higher probability of being classified as having high dietary diversity. However, the educational status of being unable to read and write was significantly associated with a lower probability of being classified as having high dietary diversity. Even though frequent consumption of okra was not associated with the dietary diversity of pregnant women, increasing the production and consumption of okra might help in food fortification and alleviation of problems associated with micronutrients such as iron and folate deficiencies which increases the level of hemoglobin concentration among pregnant women in resource-limited settings.

Therefore, the promotion of ANC follow-up and nutritional counseling on the use of a diversified diet, healthy eating behavior during pregnancy to improve their micronutrient intake (iron), and changing their food intake (dietary modification) was very important. In line with this, increasing diversified agricultural productivity and promoting frequent use of locally available wild edible plants (okra) to maintain household food security are also very crucial. Furthermore, encouragement of women to attend formal education to get nutritional knowledge and awareness was recommended in resource-limited settings as well.

## Data Availability

Data generated by our research that support our article will be made available as soon as possible, upon request wherever legally and ethically possible.
